# Routine biomarkers are strong predictors of short term mortality

**DOI:** 10.1186/1757-7241-23-S1-A55

**Published:** 2015-07-16

**Authors:** Michael Kristensen, Charlotte Barfod, Jacob Forberg, György Söletormos, Morten Schou, Kasper Iversen

**Affiliations:** 1 Department of Cardiology, Nephrology and Endocrinology, Hillerød University Hospital, Hillerød, Denmark; 2 Department of Anaesthesia, Head and Neck Surgery Centre, University Hospital Rigshospitalet, Copenhagen, Denmark; 3 Department of Emergency Medicine, Hillerød University Hospital, Hillerød, Denmark; 4 Department of Clinical Biochemistry, Hillerød University Hospital, Hillerød, Denmark; 5 Department of Cardiology, Hillerød University Hospital & Herlev University Hospital, Hillerød, Denmark

## Background

Today most patients have a routine biochemical screening taken on arrival to the emergency department. However, the results are not used in the initial assessment of the patients. Including the routine biochemical screening in a triage model may add important predictive value to the initially performed risk stratification.

## Methods

A prospective observational cohort study of 6,279 consecutive patients admitted from the Emergency Department of Hillerød University Hospital. All triaged patients with a full biochemical screening (albumin, creatinine, CRP, haemoglobin, Lactate dehydrogenase, leukocytes, potassium, and sodium) were included. Vital status was collected from the Danish Central Office of Civil Registration. The primary endpoint was 30-day mortality. Secondary endpoints were admission to intensive care unit (ICU) and readmission. Univariate logistic regression splines were made for all eight biomarkers with cuts defined after internationally accepted reference intervals. These models were used to create a multivariate logistic regression spline including all eight biomarkers, and discriminative ability was evaluated with receiver operation characteristics and area under the curve (AUC). Ultimately predicted risks of mortality based on the biomarkers were calculated for all patients, and they were divided into four groups: Green <1%, yellow 1-10%, orange 10-25%, red >25%.

## Results

5,371 patients were included (85.5%). Average age was 60.9 years [60.6; 61.1] with 48.0% [46.7; 49.3%] males. Overall mortality was 5.3% [4.7; 5.9%]. Mortality in the least acute category was 2.8% [2.0; 3.6%] for the original triage and significantly lower for the biomarker model, 0.3% [0.1; 0.5%] (p < 0.01). Mortality in the most acute triage group was 22.6% [16.1; 29.1%] and significantly higher for the biomarker model, 43.3% [37.1; 49.5%] (p < 0.01). Triage was a weak predictor of short-term mortality and demographics (age and sex) alone proved significantly stronger (AUC = 63.82% vs. 75.22%, p < 0.001). Routine biomarkers were strong predictors (AUC = 86.41%) and could improve the original triage significantly (ΔAUC = 23.66%, p < 0.001).

Biomarkers were weak predictors of admission to Intensive Care Units compared to the triage (AUC = 68.66% vs. 73.15%), however added to the triage, discrimination was improved significantly (ΔAUC = 4.75%, p < 0.001). None of the models proved able to predict re-admissions.

## Conclusion

Adding biomarkers to the presently used triage model can add significant discriminative value and improve early risk stratification of patients in the emergency department.

